# Improved *k*-*t* PCA Algorithm Using Artificial Sparsity in Dynamic MRI

**DOI:** 10.1155/2017/4816024

**Published:** 2017-07-18

**Authors:** Yiran Wang, Zhifeng Chen, Jing Wang, Lixia Yuan, Ling Xia, Feng Liu

**Affiliations:** ^1^Department of Biomedical Engineering, Zhejiang University, Hangzhou 310027, China; ^2^Center for Brain Imaging Science and Technology, Department of Biomedical Engineering, Zhejiang University, Hangzhou, Zhejiang, China; ^3^State Key Lab of CAD&CG, Zhejiang University, Hangzhou, Zhejiang, China; ^4^School of Information Technology and Electrical Engineering, The University of Queensland, Brisbane, QLD, Australia

## Abstract

The *k*-*t* principal component analysis (*k*-*t* PCA) is an effective approach for high spatiotemporal resolution dynamic magnetic resonance (MR) imaging. However, it suffers from larger residual aliasing artifacts and noise amplification when the reduction factor goes higher. To further enhance the performance of this technique, we propose a new method called sparse *k*-*t* PCA that combines the *k*-*t* PCA algorithm with an artificial sparsity constraint. It is a self-calibrated procedure that is based on the traditional *k*-*t* PCA method by further eliminating the reconstruction error derived from complex subtraction of the sampled *k*-*t* space from the original reconstructed *k*-*t* space. The proposed method is tested through both simulations and in vivo datasets with different reduction factors. Compared to the standard *k*-*t* PCA algorithm, the sparse *k*-*t* PCA can improve the normalized root-mean-square error performance and the accuracy of temporal resolution. It is thus useful for rapid dynamic MR imaging.

## 1. Introduction

High spatial and temporal resolutions are very important in dynamic magnetic resonance imaging (dMRI) clinical applications, such as functional MRI, cardiac cine imaging, and perfusion imaging among others. To improve the imaging speed of dMRI, a number of fast imaging techniques have been developed taking advantage of relevant correlations in both spatial *k*-space location and temporal dimension [1, 2]. Examples of these approaches include dynamic parallel imaging [3, 4] and several *k*-*t* methods [5, 6]. Dynamic parallel imaging techniques such as temporal sensitivity encoding (TSENSE) [3] and temporal generalized autocalibrating partially parallel acquisitions (TGRAPPA) [4] are based on time-interleaved sampling pattern and the autocalibration of full *k*-space without extra reference lines. These methods have been adopted for cardiac MR imaging [7–9]. The *k*-*t* techniques, including *k*-*t* Broad-use Linear Acquisition Speed-up Technique (BLAST)/SENSE [5] and *k*-*t* GRAPPA [6], can dramatically reduce the scan time with either multichannel or single-channel data [10, 11]. *k*-*t* GRAPPA utilizes the spatiotemporal correlations to linearly interpolate the missing data in *k*-*t* space. The *k*-*t* BLAST unfolds the signal aliasing by an adaptive filter derived from the estimated signal covariance of the low resolution data. As an enhancement of *k*-*t* BLAST, *k*-*t* principal component analysis (*k*-*t* PCA) [12] further exploits the relevant signal correlations as the temporal basis functions tailored to the training data and makes the reconstruction problems inherently overdetermined. The most fundamental assumption in *k*-*t* PCA is that the true *x*-*f* data can be represented by the defined basis functions. Consequently, it has improved temporal resolution and owns more suitable applications than *k*-*t* BLAST [13–15].

All the above-mentioned techniques can only improve the spatiotemporal resolution of MRI to certain extent; when the reduction factor goes higher, these methods usually encounter problems associated with noise amplification and residual aliasing artifacts [16–18]. Thus, new methods are called for development. For example, artificial sparsity has recently been used to improve the imaging performance [18–24]. As shown in [18], a novel approach is presented to decrease the *g*-factor in TGRAPPA, which separated the localized dynamic regions from the composite image for reconstruction only. This sparse approach is also performed on TSENSE [19]. As it improved the solution of least square function, sparse TSENSE approach could obtain robust reconstruction at high frame rates. For *k*-*t* BLAST/SENSE and *k*-*t* GRAPPA with the residual *k*-space method, in which the temporal invariant terms were calculated separately and subtracted from each frame before reconstruction, artificial sparsity was also successfully developed for dMRI applications [5, 6]. In another artificial-sparsity-based work [20], a high-pass filter was used to suppress the low frequency parts while preserving the high frequency information for GRAPPA reconstruction, which corresponded to the image details and edge information. These approaches achieved a good reconstruction quality for MR imaging with high reduction factors. In MR angiography, both contrast-enhanced and non-contrast-enhanced MR angiography [21, 22] techniques were also beneficial from artificial sparsity. It was shown that the noise amplification at high reduction factor was reduced, since the difference data denoted sparse dataset. As for high spatiotemporal resolution dynamic contrast-enhanced reconstruction, *k*-*t* ARTS-GROWL scheme was proposed to combine dynamic artificial sparsity with non-Cartesian parallel imaging [23], which results in satisfactory image quality with high computational efficiency.

In this work, we propose an approach to combine *k*-*t* PCA with artificial sparsity, termed sparse *k*-*t* PCA, for further improving the image reconstruction quality of dMRI. This new method attempts to eliminate the reconstruction error of the traditional *k*-*t* PCA algorithm in the form of the difference data between the sampled *k*-*t* space and the original reconstructed *k*-*t* space. The method is tested by using both numerical simulations and in vivo dataset with different reduction factors. Moreover, another artificial sparsity scheme named residual *k*-*t* PCA is also generated in this work, which is following the scheme in [5, 6]. Comparison between sparse *k*-*t* PCA, standard *k*-*t* PCA, and residual *k*-*t* PCA is presented.

## 2. Theory

### 2.1. A Brief Review of *k*-*t* PCA and Artificial Sparsity

**Figure 1:**
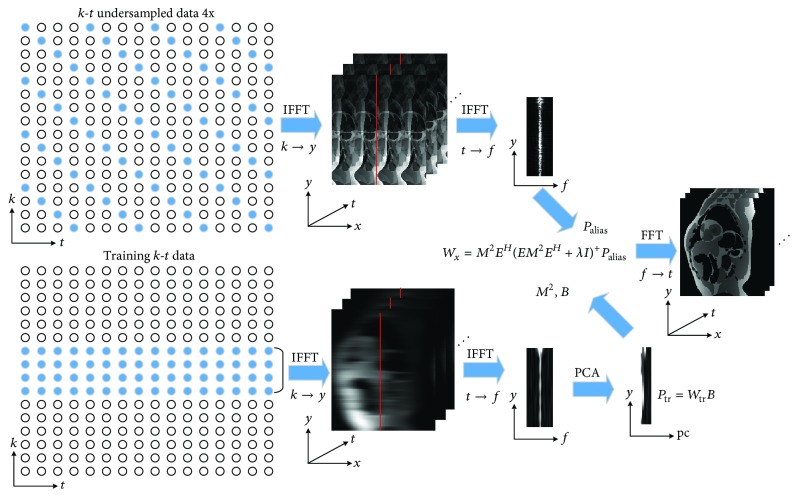
The flow chart of conventional *k*-*t* PCA reconstruction.

Figure 1 shows the workflow of *k*-*t* PCA technique [12]. The *k*-*t* PCA is a variation of *k*-*t* BLAST that further extracts the spatiotemporal correlations in the low resolution data utilizing principal component analysis (PCA) method. Based on the partially separable theory [25], it efficiently decomposes the training and undersampled signals with the use of spatial invariant basis functions and time-invariant weighting coefficients in the *x*-pc domain (*x* = spatial position, pc = principle components obtained by applying a PCA operator):

(1) $$\begin{array}{c} P_{\text{train}}=W_{\text{train}}B, \end{array}$$

where **P**_train_ is the training dataset corresponding to low spatial resolution and full temporal resolution image. **B** contains spatial invariant basis functions and the rows of **B** contain the principal components (PCs). **W**_train_ contains the weighting coefficients of the PCs for a specific spatial location in the training dataset. It is assumed that the true *x*-*f* data **P** can be decomposed by the defined basis functions **B** as follows:

(2) $$\begin{array}{c} P=WB, \end{array}$$

where **W** is the *x*-pc representation of **P** and the rows of **W** contain the spatial location *x*. The aliased signal intensity at location (*x*, *f*_*m*_) is given by the sum of *R* (acceleration factor) pixels in true *x*-*f* voxels depending on the sampling point spread function:

(3) $$\begin{array}{c} P_{\text{alias}}\left(\;x,f_{m}\;\right)=\left[\begin{array}{cccc} 1 & 1 & \cdots & 1 \end{array}\right]\left[\begin{array}{c} P\left(x_{1},f_{m,1}\right)\\ P\left(x_{2},f_{m,2}\right)\\ \vdots \\ P\left(x_{R},f_{m,R}\right) \end{array}\right], \end{array}$$

where $\left[\begin{array}{cccc} 1 & 1 & \cdots & 1 \end{array}\right]$ represents the unit row vector and the length is *R*. (*x*_*i*_, *f*_*m*,*i*_) indicates the position of *i*th aliasing pixel in the true object.

In (2), the weighting coefficient matrix **W** is temporally invariant, the aliased signal of all temporal frequencies at location *x* can be collected in a vector **P**_alias,*x*_, and the unknown spatial weighting at aliasing positions can be collected in the vector **W**_*x*_. Then an encoding matrix **E** is introduced, which contains the spatially invariant basis function **B**, as follows:

(4) $$\begin{array}{c} P_{\text{alias},x}=EW_{x}. \end{array}$$

Exploiting the Tikhonov regularized least-squares solution to the above Equation (4), **W**_*x*_ is given as follows [12]:

(5) $$\begin{array}{c} W_{x}=M^{2}E^{H}{\left(\;EM^{2}E^{H}+\lambda I\;\right)}^{+}P_{\text{alias},x}, \end{array}$$

where *M*^2^ = diag⁡(*w*_train,*x*_*w*_train,*x*_^*H*^) is the signal covariance matrix. The superscript *H* is the conjugate transpose operator, and superscript + represents the Moore-Penrose pseudoinverse. **I** denotes the identity matrix, and *λ* is the regularization parameter to balance the fidelity of the solution and Tikhonov regularization. Larger values of *λ* will smooth the solution, while small values bring high noise level.

Artificial sparsity is a useful concept to be used to improve the performance of the parallel imaging or parallel imaging like approaches [5, 6, 18–24]. For parallel imaging, better reconstruction accuracy can be achieved when the image content becomes artificially sparse, because there are fewer pixels superimposed on each other. For *k*-*t* PCA, when the acceleration factor goes higher, the condition of the encoding matrix *E* is worse and may suffer from noise amplification and higher reconstruction error. The fundamental assumption underlying sparse *k*-*t* PCA is that *k*-*t* PCA will have better performance when the image support reduces and the sparse data will improve the condition of the encoding matrix inversion.

Because artificial sparsity and *k*-*t* PCA use fundamentally different acceleration methods, it is feasible to combine them together for a better performance in this work.

## 3. Materials and Methods

### 3.1. Flowchart of Residual *k*-*t* PCA and Sparse *k*-*t* PCA

**Figure 2:**
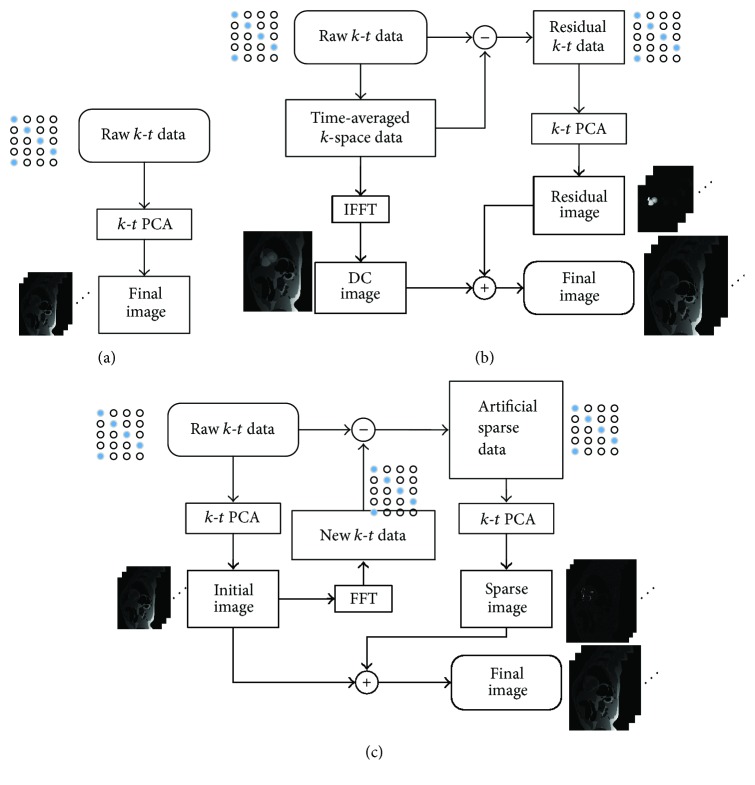
Flow charts of standard *k*-*t* PCA (a), residual *k*-*t* PCA (b), and sparse *k*-*t* PCA (c). (The concept of “New *k*-*t* data” is the undersampled *k*-space data corresponding to the raw *k*-space locations.)

Figure 2 shows the flow charts of standard *k*-*t* PCA and two variants of *k*-*t* PCA reconstructions proposed in this work, which is demonstrated using the cardiac perfusion phantom data.  Figure 2(b) shows *k*-*t* PCA combined with a sparse processing, termed residual *k*-*t* PCA in this work. Its procedure is summarized as follows: (1) firstly, the inverse Fast Fourier Transform (IFFT) is conducted on the time-averaged *k*-space to obtain the direct-current (DC) image; (2) this time-averaged *k*-*t* space is then subtracted from each frame of the sampled *k*-*t* data to get the residual *k*-*t* space; (3) after the *k*-*t* PCA reconstruction, the final image is constructed by adding the DC image and residual image together.

The procedure of the proposed method is summarized in Figure 2(c): (1) apply traditional *k*-*t* PCA to obtain the original image; (2) after Fast Fourier Transform (FFT), the corresponding *k*-*t* space is gained according to an undersampling pattern; (3) by complex subtracting the sampled *k*-*t* space from the corresponding *k*-*t* space, the sparse data is artificially produced; (4) then, take *k*-*t* PCA reconstruction to generate the sparse image; (5) finally, add the sparse image into the original reconstructed result to obtain the final image. In sparse *k*-*t* PCA method, the procedure of *k*-*t* PCA introduces the reconstruction error inevitably. If this error information is well restored, the final reconstruction will be improved.

### 3.2. Numerical Simulations

To validate the presented *k*-*t* method against the reference, a fully sampled 2D image was simulated using the MRXCAT first-pass myocardial perfusion numerical phantom [26] with default settings unless explicitly stated. The relevant imaging parameters of the simulated perfusion dataset included pulse repetition time (TR)/echo time (TE) = 2/1 ms; flip angle (FA) = 15°; spatial resolution = 2 × 2 × 5 mm^3^, matrix size = 224 × 192. No respiratory motion was involved in the dataset. The dose of contrast agent was 0.075 mmol/kg. This phantom dataset was made up of 32 time frames and collected by four receiver coils. Normalized-distributed complex white Gaussian noise was then added to the phantom data for all the coils to yield a signal-to-noise ratio (SNR) of 20 dB. The simulation data were undersampled and reconstructed using standard, residual, and sparse *k*-*t* PCA. Specially, the simulation data consisted of two separate parts: training data and undersampled data. The training data were selected from a center region of *k*-space in each time frame, despite not being used in the final reconstruction. The undersampled data were obtained using an interleaved sampling pattern on the sheared-grid [12]. The reduction factor indicated the degree of accelerated sampling in the undersampled *k*-*t* space. To compare the results from different reconstructions, all the *k*-*t* PCA methods were performed using 11 training profiles with the reduction factors from 4 to 8.

### 3.3. In Vivo Experiments

One volunteer participated in the study. The fully sampled in vivo experiment of 2D first-pass cardiac perfusion MR imaging was performed on a 3.0 Tesla MR system (Tim Trio; Siemens Healthcare, Erlangen, Germany), with 12 channels of both body flexible coils and spine coils. The dataset was acquired using ultrafast spoiled gradient echo sequence with the following parameters: TR/TE = 2.5/1.3 ms; FA = 10°; field of view = 320 × 320 mm^2^; spatial resolution = 1.67 × 1.67 mm^2^; matrix size = 192 × 192 and the time frames = 40. Data acquisition was initiated simultaneously with intravenous injection gadopentetate dimeglumine (Magnevist, Bayer Healthcare, Leverkusen; 0.1 mmol/kg at 6 mL/s) followed by a 20 mL saline flush, injected at a rate of 2 mL/s. The training data and undersampled data were yielded as in the numerical simulations, and all the in vivo experiments were also reconstructed empirically with 11 training profiles. All human studies were conducted under Institutional Review Board approval. The participant signed the Informed consent before the imaging experiments.

All the datasets were reconstructed offline in the MATLAB commercial software (version: R2014a; the Mathworks Inc., Natick, MA). During the processing of all the *k*-*t* PCA methods, the number of principal components was investigated and chosen to optimize the normalized root-mean-square error (NRMSE) in each case. In this work, the number of principal components was empirically chosen to be 6 in numerical simulation and 7 in in vivo experiments. Furthermore, all the multichannel data was firstly reconstructed channel by channel and then combined with the sensitivity maps for the optimum SNR according to [12]

(6) $$\begin{array}{c} I=\overset{N_{c}}{\underset{j=1}{\sum }}I_{j}\cdot S_{j}^{*}. \end{array}$$

Here *S*_*j*_ is the coil sensitivity map corresponding to coil *j* and *N*_*c*_ is the total coil counts.

### 3.4. Image Analysis

Both the simulations and the in vivo datasets use the absolute error maps to accurately visualize the reconstruction, which are calculated by subtracting the reconstruction images from the fully sampled image.

For quantitative evaluation, the reconstruction quality of the numerical simulation is also evaluated by NRMSE and mean-NRMSE (*m*-NRMSE). The NRMSE reports the reconstruction errors relative to the reference image for each case by

(7) $$\begin{array}{c} \text{NRMSE}=\frac{{\left\|I_{\text{ref}}\left(r\right)-I_{\text{rec}}\left(r\right)\right\|}_{F}}{{\left\|I_{\text{ref}}\left(r\right)\right\|}_{F}}, \end{array}$$

where **I**_rec_ is the reconstructed image from undersampling and **I**_ref_ is the reference image corresponding to the full sampled image. *F* represents the Frobenius norm.

*m*-NRMSE represents the averaged NRMSE across all time frames that can be expressed as

(8) $$\begin{array}{c} m\text{-NRMSE}=\frac{\sum_{t=1}^{N}{\text{NRMSE}}_{t}}{N}, \end{array}$$

where NRMSE_*t*_ is the NRMSE corresponding to the reconstructed image of frame *t* and *N* is the total frame counts.

The *g*-factor and SNR maps are calculated using a pseudo multiple replica approach [27]. The number of replicas is set to 200 in this work.

In temporal resolution evaluation, the signal-intensity time courses of two manually drawn regions of interest (ROI) are plotted to assess the perfusion reconstruction results, that is, the left and right ventricles.

## 4. Results

### 4.1. Numerical Simulations

**Figure 3:**
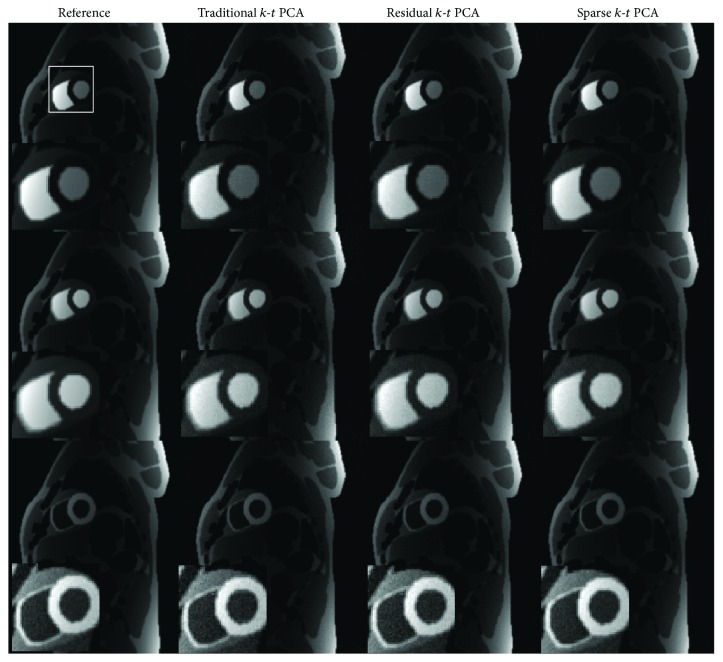
Results of numerical simulations with reduction factor of 4 using three different reconstruction methods. The 9th frame in the time sequence corresponds to Row 1, the 13th frame corresponds to Row 2 and the 25th frame corresponds to Row 3. The small figures in the left bottom corner of each figure demonstrate the corresponding enlarged region defined by the white box. The reconstructed images are shown in the same intensity scale. The reference images are reconstructed by directly applying FFT for fully sampled data.

**Figure 4:**
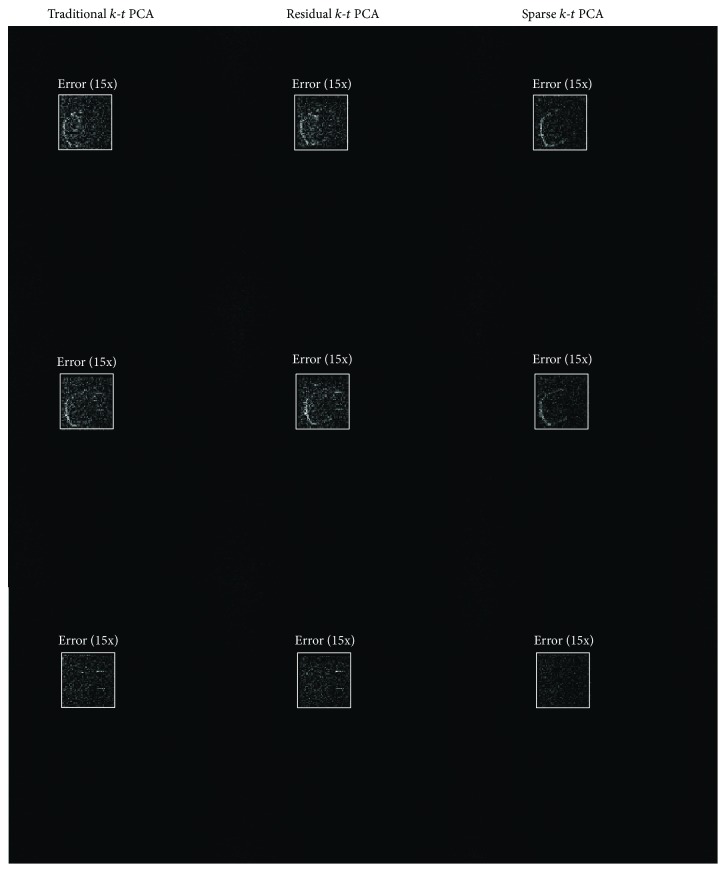
Absolute error maps between the reference images and the corresponding reconstructed images in Figure 3. The three columns correspond to traditional, residual, and sparse *k*-*t* PCA from left to right, respectively. The ROI defined by the white box in each figure was brightened 15 times for better visualization.

**Figure 5:**
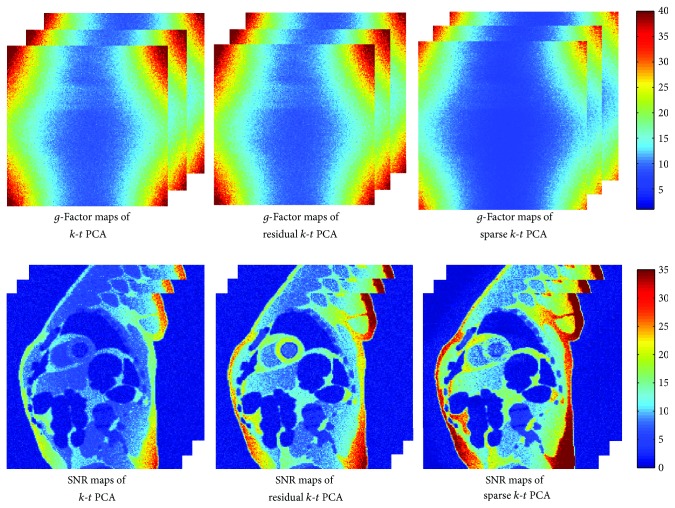
Comparison of *g*-factor and SNR maps of different approaches.

**Figure 6:**
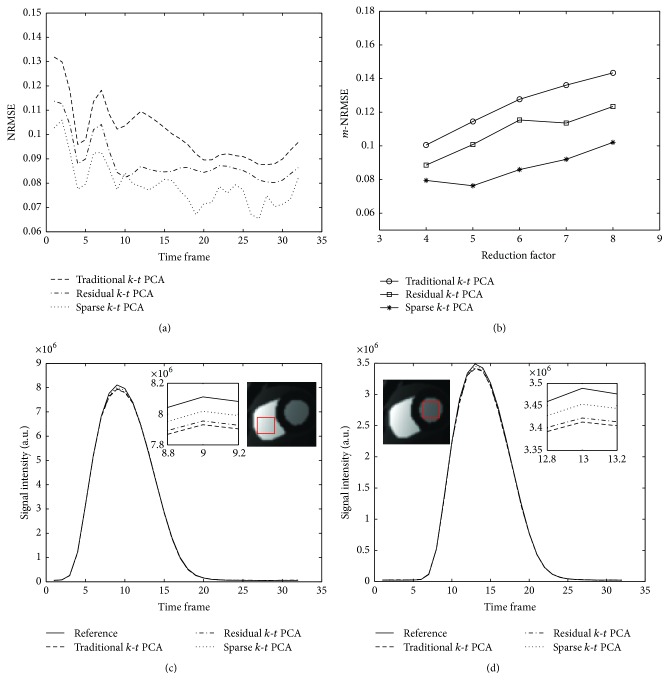
Summary of the quantitative performance of three *k*-*t* PCA methods on numerical simulations. The plots show the NRMSE of different time frames of 4-fold acceleration (a), *m*-NRMSE of different reduction factors (b), and the signal-intensity time courses of left ventricle (d) and right ventricle (c) at the reduction factor of 4 with different time frames.

Figures 3, 4, 5, and 6 show the results of the cardiac perfusion phantom.  Figure 3 shows the results of three temporal frames with the reduction factor of 4 from different *k*-*t* PCA methods. When looking into the zoomed in figures, it can be seen that the images reconstructed by sparse *k*-*t* PCA contain less noise than the other *k*-*t* PCA reconstructions for all the displayed frames.  Figure 4 shows the absolute error maps of the three corresponding different *k*-*t* PCA images in Figure 3. From the brightened ROI of the error maps, we can see less observable noise and reconstruction error generated from sparse *k*-*t* PCA compared to the results from traditional and residual *k*-*t* PCA approaches.

Figure 5 displays *g*-factor and SNR maps of all the tested approaches in this work. From this figure, we can see lower *g*-factor and higher SNR benefit in sparse *k*-*t* PCA compared to other approaches used in this article. Compared to the standard *k*-*t* PCA, the *g*-factor and SNR improvements are obvious.

Figure 6 summarizes the quantitative performance of the numerical simulations.  Figure 6(a) shows the image quality related results (NRMSE) of 4-fold acceleration. The plots in Figure 6(a) highlight the improvement of NRMSE of sparse *k*-*t* PCA at all the time frames. The mean-NRMSE (*m*-NRMSE) values of the traditional, residual, and sparse *k*-*t* PCA methods are 10.1%, 8.9%, and 7.9% individually.  Figure 6(b) displays the *m*-NRMSE values of different reduction factors; sparse *k*-*t* PCA always shows better *m*-NRMSE than other tested approaches. Figures 6(c) and 6(d) illustrate the signal-intensity time courses derived from the ROI placed in the right ventricle and left ventricle, respectively. Compared to the reference signal-intensity time courses (the solid lines in Figures 6(c) and 6(d)), all the *k*-*t* PCA methods show good resemblance while the sparse *k*-*t* PCA reconstruction is observed with better accuracy at the peak of the curve. In conclusion, the sparse *k*-*t* PCA reconstruction approach shows less prone to error and has higher NRMSE than the traditional and residual *k*-*t* PCA at all the temporal frames.

### 4.2. In Vivo Experiments

**Figure 7:**
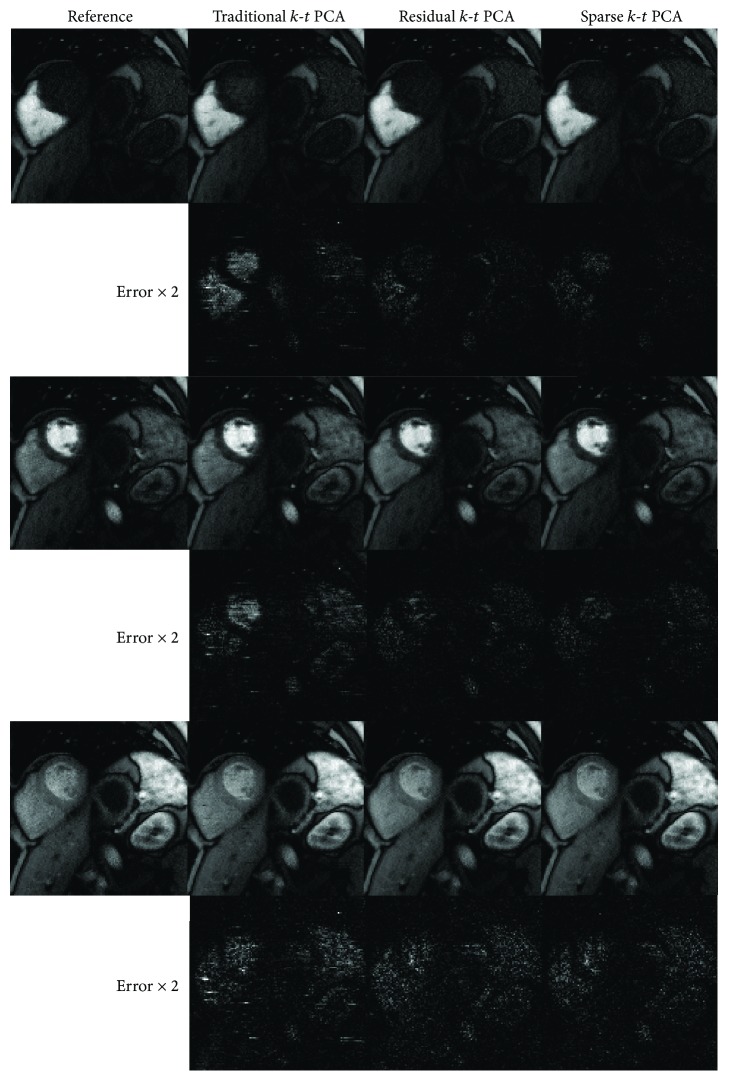
The 4-fold accelerated in vivo results at the selected frames and the corresponding absolute error maps are shown in the following rows, reconstructed by traditional, residual, and sparse *k*-*t* PCA approaches. The reconstructed images are shown in the same intensity scale, and the error maps are scaled twofold for better visualization. The reference images are reconstructed by applying FFT for fully sampled data.

Figure 7 shows the 4-fold accelerated in vivo reconstructions of the cardiac perfusion at three time frames: 8th, 14th, and 22nd, respectively. The absolute error maps are displayed together with the reconstructed result, which prove lower reconstruction error in the sparse *k*-*t* PCA reconstructions compared to other tested approaches.

Figure 7 shows some reconstructed frames and the corresponding absolute error maps of the 4-fold accelerated in vivo cardiac perfusion dataset. Compared with conventional *k*-*t* PCA, the absolute error maps of sparse *k*-*t* PCA show fewer artifacts and visible errors around the heart region. Compared to residual *k*-*t* PCA, the result of the sparse reconstruction method also looks slightly better.

**Figure 8:**
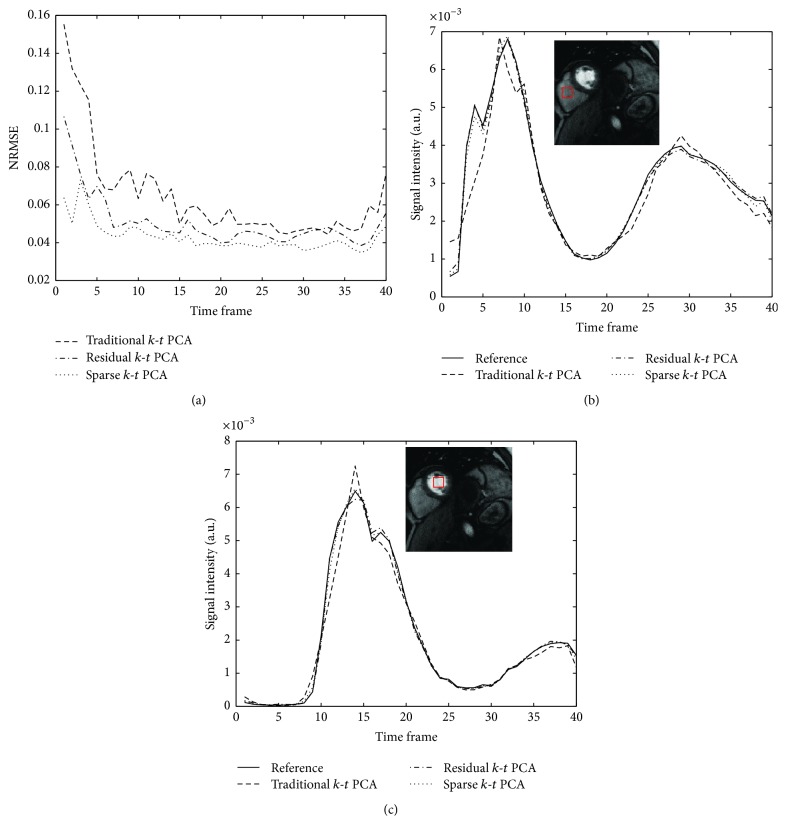
Summary of the quantitative performance of three *k*-*t* PCA methods on 4-fold accelerated in vivo dataset. The plots show the NRMSE (a) and the signal-intensity time courses of two manually drawn ROIs (b, c) at the reduction factor of 4 with different time frames.

Figure 8 shows the NRMSE curves of all the temporal frames and the signal-intensity time courses of two manually selected ROIs.  Figure 8(a) demonstrates that the NRMSE criterion of sparse *k*-*t* PCA is the best among all the tested algorithms. In this in vivo experiment, the *m*-NRMSE values are 6.5%, 5.1%, and 4.3% for the traditional, residual, and sparse *k*-*t* PCA methods, respectively. Figures 8(b) and 8(c) display the signal-intensity time courses of two manually drawn ROIs (two ventricles). Sparse *k*-*t* PCA and residual *k*-*t* PCA yield better consistency with the reference than traditional *k*-*t* PCA. Overall, it is demonstrated that the sparse *k*-*t* PCA generates lower reconstruction errors than other tested approaches in this work.

## 5. Discussion

Based on the numerical simulations and in vivo data, the sparse *k*-*t* PCA reconstruction scheme is proposed to compare with traditional *k*-*t* PCA and residual *k*-*t* PCA in this paper. This is accomplished by correspondingly complex subtracting the sampled *k*-*t* space from the once reconstructed *k*-*t* space, and then add the processed difference images back into the original results. Both the simulation and in vivo experiments have shown the improved image quality and reduced NRMSE values in this work.

It has been shown that some methods, such as parallel imaging, could be expected to achieve better reconstruction quality when the image support becomes smaller, because they inherently incorporate the sparse image content via the coil sensitivity profile. In the proposed method, the sparsity of the difference data can also be exploited to improve the *k*-*t* PCA reconstructions from the weighting calculation. The reconstruction of original *k*-*t* PCA is supposed to improve, as the error information can be accurately reproduced and removed through the artificial sparse reconstruction.

The observations made from the numerical simulations are also verified in the in vivo experiments. When the acceleration factor is 4, compared to traditional *k*-*t* PCA, the *m*-NRMSE of the presented sparse *k*-*t* PCA has reduced by 22% for numerical data and 34% for in vivo data, respectively. From the comparisons in Figures 3, 4, 6, and 7, we are able to see lower artifacts and noise level in the sparse reconstruction images than standard *k*-*t* PCA. It means that sparse *k*-*t* PCA can enable a self-calibration effect on traditional *k*-*t* PCA. When the reduction factor becomes higher, the *m*-NRMSE of sparse *k*-*t* PCA is still better than the other two *k*-*t* PCA approaches (Figure 6(b)), and the improvement over the traditional *k*-*t* PCA is obvious. For temporal resolution, the signal-intensity time courses (Figures 6(c), 6(d), 8(b), and 8(c)) also prove the superior performance of the sparse *k*-*t* PCA.

Although reconstructions from our undersampled data demonstrate the feasibility of the presented framework, it still needs some investigations in practical experiments. When the reduction factor goes higher, the reproduction of error information should be investigated in sparse *k*-*t* PCA for better reconstruction of dynamic signal. In addition, this kind of scheme is performed on 2D dynamic imaging using *k*-*t* PCA; it can also be extended to other *k*-*t* parallel imaging type approaches (e.g., *k*-*t* BLAST/SENSE [5], *k*-*t* GRAPPA [6], and *k*-*t* SPIRiT [28]) for further study.

## 6. Conclusions

The improved *k*-*t* PCA method obtains higher reconstruction quality compared with traditional one for the tested imaging cases. Compared to the residual *k*-space method, sparse *k*-*t* PCA always achieves better performance. The experimental results suggest that sparse *k*-*t* PCA can improve the clinical applicability of dynamic MRI.
